# Depressive Symptoms among Industrial Workers in Vietnam and Correlated Factors: A Multi-Site Survey

**DOI:** 10.3390/ijerph16091642

**Published:** 2019-05-11

**Authors:** Bach Xuan Tran, Giang Thu Vu, Kiet Tuan Huy Pham, Quan-Hoang Vuong, Manh-Tung Ho, Thu-Trang Vuong, Hong-Kong T. Nguyen, Cuong Tat Nguyen, Carl A. Latkin, Cyrus S.H. Ho, Roger C.M. Ho

**Affiliations:** 1 Institute for Preventive Medicine and Public Health, Hanoi Medical University, Hanoi 100000, Vietnam; phamhuytuankiet_vkt@fpt.vn; 2 Bloomberg School of Public Health, Johns Hopkins University, Baltimore, MD 21205, USA; carl.latkin@jhu.edu; 3 Center of Excellence in Evidence-based Medicine, Nguyen Tat Thanh University, Ho Chi Minh City 70000, Vietnam; giang.coentt@gmail.com (G.T.V.); pcmrhcm@nus.edu.sg (R.C.M.H.); 4 Centre for Interdisciplinary Social Research, Phenikaa University, Yen Nghia, Ha Dong, Hanoi 100803, Vietnam; hoang.vuongquan@phenikaa-uni.edu.vn (Q.-H.V.); tung.homanh@phenikaa-uni.edu.vn (M.-T.H.); 5 Faculty of Economics and Finance, Phenikaa University, Yen Nghia, Ha Dong, Hanoi 100803, Vietnam; 6 Sciences Po Paris, Campus de Dijon, 21000 Dijon, France; thutrang.vuong@sciencespo.fr; 7 A.I. for Social Data Lab (AISDL), Vuong & Associates Co., Hanoi 100000, Vietnam; htn2107@caa.columbia.edu; 8 Institute for Global Health Innovations, Duy Tan University, Da Nang 550000, Vietnam; cuong.ighi@gmail.com; 9 Department of Psychological Medicine, National University Hospital, Singapore, Singapore 119074, Singapore; cyrushosh@gmail.com; 10 Department of Psychological Medicine, Yong Loo Lin School of Medicine, National University of Singapore, Singapore 119228, Singapore; 11 Biomedical Global Institute of Healthcare Research & Technology (BIGHEART), National University of Singapore, Singapore 119228, Singapore

**Keywords:** depression, industrial workers, migrant workers, Vietnam

## Abstract

Depressive disorders have been found to be a significant health issue among industrial workers, resulting from work-related factors and causing serious burdens for the workers as well as their employers. Literature on mental health problems of Vietnamese industrial workers has been limited, despite the rapid foreign investment-fueled industrialization process of the country. This study aimed to fill the gap in literature by examining the prevalence of depressive disorders and their potential associated factors among a cohort of Vietnamese industrial workers. A cross-sectional study was conducted in 3 industrial areas in Hanoi and Bac Ninh. A total of 289 workers agreed to participate in the study. Generalized linear mixed models were applied to identify associated factors with depression status of respondents. 38.6% of participants reported suffering depression. Factors found to be positively associated with the possibility of having depression and higher PHQ9 score were long working hours, suffering more health problems, and health hazards exposure at work. Meanwhile, being females, having more children, living with parents, engaging in smoking, and being local workers were found to correlate with lower likelihood of being depressed. The findings suggested the importance of regular health screening, work safety assurance, and social support outside of workplace on the mental health of the workers.

## 1. Introduction

The World Health Organization (WHO) has classified common mental disorders, which include depressive and anxiety disorders, as “neurotic, stress-related, and somatoform disorders” and “mood disorders” [1]. These mental and behavioral disorders carry major weight in public health, as they are among the leading source of disease burden globally [2,3,4]. The burden of mental disorders on occupational health is particularly significant, since these disorders can lead work-related outcomes like absenteeism, sub-optimal performance at work, and high rate of turnover [5] which negatively affect both the workers and the employers. In developing countries, the previously neglected issue of psychological health of employees, especially of those working as industrial workers, has been brought into focus in recent years, amidst the rapid increase of the number of industrial factories established on foreign investment. 

The relationship between the workplace and workers’ mental health is a complex one [6]. While work itself can elevate an individual’s well-being through its sense of job security, social contact, and organizational skills [7], a stress-inducing working environment could cause adverse mental health for the workers, which would in turn decrease their productivity and disrupt work [6,8,9]. A systematic review of research on work environment and depressive symptoms by Theorell, Hammarström [9] showed a substantial amount of empirical evidence for employees, both male and female, who lack control and face job strain and bullying to be at higher risks of depressive symptoms over time. Similarly, when looking at heavy industry workers who face high demand and have moderate control, Cantley, Cullen [8] found a higher risk of self-reported depression among these individuals. A number of studies have confirmed that industrial workers who reported high job strains and low social support to be suffering from depression and/or psychosocial distress, which includes social dysfunction, anxiety, and insomnia [10,11]. Meanwhile, a study on Latino manual laborers found that those who reported neck and shoulder pain were also more likely to present depressive symptoms [12].

Studies on factors associated with depression and anxiety in the general population have listed noise annoyance [13,14], air pollution [15,16,17,18], and elevated levels of environmental neurotoxicants [19,20] as the outstanding elements. Translating to workplace environment, it is expected that these stressors would affect the mental health state of workers in some extent. In a different example, aircraft maintenance workers who are exposed to long-term low-level organophosphate esters tend to self-report depression [21]. Workplaces that are plagued by prolonged industrial relations conflict such as regular factory strikes may also increase the stress for workers, thereby driving up the likelihood of anxiety and depression [22]. For a large part, little is known about the accumulation of various factors in the industrial work environment that may be related to depression or other types of mentor disorder.

On the risk factors for depression, evidences from both developing and developed countries have shown depression to be associated with the female sex, especially those who suffer from severe life events, such as relationship crises, deaths, fertility or unwanted pregnancy, and lack social support in these circumstances [23,24,25,26,27]. In particular, women are about twice as likely as men to develop depression during their lifetime – an alarming gender gap that could be attributed to biological, psychological, and environmental factors [27]. Albert [26] suggested that “the differential risk may primarily stem from biological sex differences and depend less on race, culture, diet, education, and numerous other potentially confounding social and economic factors.” 

To the best of our knowledge, there has been a lack of study on the factors associated with depressive illness in industrial workers in Vietnam, especially in the recent context of increased foreign investment and industrialization [28]. In fact, the subject of mental health disorders in Vietnam has still been inadequately researched—to date we have found only one national-wide study conducted in 2001–2003 period that covered the topic, which reported the prevalence of depression among the Vietnamese population to be 2.8% [29], much lower than the figures reported for Japan (18%), Iran (14.4%), or China (13.2%) [30,31]. To fill the gap, this study examined the exposure of Vietnamese workers at factories to factors correlated with depressive symptoms, situating the empirical findings within the bunk of international research on mental health of industrial workers in resource-scarce settings.

## 2. Materials and Methods 

### 2.1. Study Design

A cross-sectional study was conducted from July to September 2018 in 3 industrial areas in Hanoi and Bac Ninh, two provinces with the most developed industrial zones in Northern Vietnam. In these areas, companies are manufacturing electronic components, vehicle accessories, automotive electronics, home electronics, telecommunications equipment, and control devices. 

We approached respondents conveniently at their living areas by inviting them to attend a health communication meeting. We invited them to take the interview after the meeting and informed consent were given. A total of 289 workers agreed to participate in the study. 

### 2.2. Measure and Instruments

Data were collected via 20-minute face-to-face interviews conducted by trained interviewers at a small private counseling room in order to protect their confidentiality. The purpose of the study, benefits, and drawbacks when participating in the study were introduced to participants, and then they were asked to join the study. 

We conducted a pilot survey on 20 participants of different ages and genders, and only minor changes to the wording were made in order to meet participants’ preferences and culture. Data concerning socio-economic characteristics, working conditions, depression level, health risk behavior, health-related quality of life, and social support situation of participants were collected, in particular: 

#### 2.2.1. Socioeconomic Characteristics

We asked participants to report their information about gender, age, education attainment, marital status, monthly income, and number of children in their family. We also collected the information regarding whether they were immigrant workers (i.e., migrated from regions of the countries rather than the current region of the factory in pursuit of work) or not. 

#### 2.2.2. Working Conditions

We asked each participants to report the following information: their years of working experience, number of working hours per day, whether they have been equipped with labor protection, and toxic factors which may cause occupational diseases that they were exposured to during working. 

#### 2.2.3. Patient Health Questionnaire-9 (PHQ-9)

To assess the levels of depression severity among participants, we used PHQ-9, a validated depression screening tool based on the Diagnostic and Statistical Manual of Mental Disorders (DSM-IV) criteria, including nine items. Each question would be pointed from 0 to 3, and the total score would be ranged from 0 to 27. Depression severity was characterized as none (0–4), mild (5–9), moderate (10–14), moderately severe (15–19), and severe (≥20). A PHQ-9 score ≥10 was seen as the cut off point for major depression with a sensitivity of 88% and a specificity of 88% [32].

#### 2.2.4. Health Risk Behavior

Substance abuse behaviors were also investigated as potential covariates. We asked participants about drinking practices, which were measured using the Alcohol Use Disorders Identification Test-Consumption-AUDIT-C included three questions and the total score that participants received was from 0 to 12. Hazardous drinking was defined if the total score is higher than 4 for males and higher than 3 for females. Smoking status was classified into three main groups: current smokers, former smokers, and never-smokers. Current tobacco status were identified if participants smoked in the last 30 days and smoked 100 cigarettes or more in their lifetime. Those who had smoked 100 cigarettes or more in their lifetime but had not smoked in the last 30 days were classified as former smokers. 

#### 2.2.5. Social Support

Attitude of participants towards social support were reported including neighbourhood cohesion [33] (positive social support), neighbourhood disorder [34], and social disorder [34] (negative social support) around the living area. Each positive answer in terms of social support counted as 1 point. Social support score ranged from 0 to 8 points.

### 2.3. Statistical Analysis

Data were analyzed by STATA version 12 (Stata Corp. LP, College Station, TX, USA). We used Chi-square test to find the differences of categorical variables between depression and non-depression group. Meanwhile, Mann–Whitney test were employed on continuous variables Generalized linear mixed models (GLMM) were applied to identify associated factors with depression status of respondent. We used GLMM to account for the potential correlation of outcomes between participants from the same factory. We also estimated robust standard errors, adjusting for the clustering within factories. The study considered any variable with a *p*-value less than 0.05 as statistically significant. 

### 2.4. Ethics approval

This study protocol was approved by the Institutional Review Board of Youth Research Institute, Hanoi, Vietnam (01a QĐ/VNCTN).

## 3. Results

Table 1 shows the prevalence of depression among our respondents to be 38.6%. Regarding socio-economic profile, 83.3% of participants were female, 51.1% were immigrants, 91.3% had high school education or above, and 95.9% lived with spouse or partner. A high proportion of participants had 2 children (64.1%). The mean age of participated worker was 31.9 years old and their monthly income was 273.6 USD per month. The mean PHQ9 score in non-depression group was 1.5 and this score in depression group was 9.1. There was statistically significant difference in terms of mean PHQ9 score between those reported not having depression and those reported having.

Table 2 demonstrates the working conditions of the participants. The average working hours per day of the participants was 8, the average years of experience was 10. There were statistically significant differences in daily working hours and number of health hazards exposure at work between depressed and non-depressed participants. Workers who had depression were more exposed to toxic gas and accident-prone hazards at workplace (*p*-value < 0.01). In contrast, those who were not depressed had more exposure health hazards relating to high temperature (*p*-value = 0.01) and toxic chemical (*p*-value = 0.05).

Table 3 indicates that 86.2% of the participants had never smoked. Percentage of people engaged in smoking and hazardous drinking were 7.3% and 13.7%, respectively. Those who had depression had more health problems than those who did not (mean number of health problems were 2.5 and 1.5 respectively, *p*-value < 0.01). 

Table 4 illustrates that people that had depression assumed that people in their living area were less willing to help each other/neighbors, less likely to be trusted and to share similar values, their living area had a tremendous amount of trash, and more social vices and fighting than was reported by people who did not have depression.

Table 5 presents the factors associated with mental health status of participants. Those who were older, had more children, and were current smokers have statistically significant lower PHQ9 score as well as lower likelihood of having depression; while those having higher years of working and suffering more health problems were statistically more likely to have higher PHQ9 score and likelihood of being diagnosed with depression. Those who were former smokers and had higher social support score have statistically significant lower PHQ9 score, while having high school education and lower as well as longer daily working hours were found to have statistically significant correlation with higher PHQ9 score. Local workers were less likely to be depressed compared to their immigrated counterparts (OR = 0.51, CI = 0.24-0.85). Females and those with hazardous drinking habits had statistically less chance of having depression. In addition, though not statistically significant, the results showed that those living with parents or children had lower likelihood of having higher PHQ9 score and suffering depression, while those living with spouse reported an opposite trend. 

## 4. Discussion

Our study found a high prevalence of depression among a cohort of Vietnamese industrial workers. Multiple concurrent health problems and exposure to hazards at work has been found to positively correlated with the likelihood of suffering depression, while gender (female) and familial supports are negatively associated factors. These findings support the implementation of regular health check-up and psychological counselings, as well as having family involvements in controlling depression and improving the well-being of workers. 

The prevalence of depression in our respondents was comparable to that reported by a number of studies on mental health of migrant workers in Australia—32.6% [35], China—34.4% [36], Peru—38.0% [37], and Spain—39.5% [38], while being almost 2.6 times higher than the figure of combined prevalence of 10 most common mental disorders in Vietnam (14.9%), found in a national survey in 2002–2003 [29]. Previous studies on Chinese industrial workers suggested that industrial workers, especially those who had emigrated to pursue work at the factories, generally had a lower level of education, worked repetitive labor-intensive jobs, were not sufficiently covered by health insurance, and were with limited family and friends support [39,40], which made them a vulnerable population with elevated risks of depression compared to other groups.

Investigating further into potential associated factors of depression among industrial workers, we found positive correlates that were also reported elsewhere: working long hours [41,42] and having more health problems [43]. On the other hand, being female was found to be negatively correlated with the likelihood of having depression, which may be considered contradictory with some research that has found women at workplace to have higher rates of depression than their male counterparts [44]. However, several studies on industrial workers indeed reported a higher proportion of male workers suffering depression than that of female workers [45] or no significant difference in gender regarding prevalence of depression [40]. Such findings may be explained by the fact that men may be more willing to take or more likely to be assigned more dangerous jobs or heavier workload, leading to longer working hours and increased work hazard exposures [45], raising their proneness to depression. With regard to our study, the sampling technique adopted may also be a reason for lower possibility of having depression in women. 

In addition, our sample showed that having more children and having higher social support score were associated with lower possibility of having depression and with lower PHQ9 score. This result is somewhat in line with existing literature that cited lack of family support, for example absence of connection with family in terms of phone calls or visiting [40], as a risk factor for depression, especially in those moved away from hometown [46]. Thus, the role and current status of social support for industrial workers, in particular those migrated from other areas, should receive more attention from company managers, community, and policy-makers. 

Albeit having a small number of participants reported as ever being smokers, our study also found that engaging in smoking, either currently or in the past, as well as hazardous drinking, was associated with lower likelihood of depression and with lower PHQ9 score. The reason for such a finding may be the fact that substance use, for instance, smoking, can be utilized as a coping mechanism for stress and depression [47], and thus smoking can arguably reduce the perceived depression symptoms among smokers [48]. Nevertheless, the impact of substance use on psychological distress, whether reducing or increasing, has still been debatable. Smoking and drinking as a potential coping mechanism for depression and work-related stress should be considered as a possibly significant health risk behavior among industrial workers, which would later result in more health issues that cause more stress and lead to depression.

Several implications can be drawn from our findings. First, regular health screening with a stronger emphasis on mental health and depression diagnosis should be conducted in all industrial factories. In addition to ensuring that the work safety requirements are being adhered to at all times to minimize work-related hazard exposures, support in a more informal and psychologically and socially-oriented form should be made available to those industrial workers. Incentives for workers to build their home in the area of the workplace, encouragement for periodical or regular visiting or connecting with their families in their hometown may be some of the suggestions for lowering the depression prevalence among industrial workers. Furthermore, regular education campaigns on healthy and health risk behaviors should be conducted to enhance the knowledge of those workers on health so that they can better manage their mental and physical condition.

The current study also has some limitations. First, the sampling method adopted may hinder the generalizability of our results. Future research should consider a larger and more diverse population to confirm the consistency of the empirical findings here. Nonetheless, the use of a structured questionnaire in this study allows for replication of research and provides a point of reference which may benefit future studies. Second, our findings may be subject to recall bias due to the self-reported nature of our survey. There were also missing responses, due to participants declining to give information when asked, reported for some questions, which may affect the interpretation of results and power of the study. However, such an effect is expected to be non-significant, since the missing rate was under 10% for all of these questions. Additionally, as a cross-sectional research, the study has not accounted for the progress of depressive symptoms and illness over time in these industrial workers.

## 5. Conclusions

Our study discovered a relatively high prevalence of depression among industrial workers. In addition, we investigated the correlation of gender, working conditions, social support, and health risk behaviors with the depression status of these workers. The findings suggested the importance of regular health screening, work safety and social support outside of workplace on the mental health of the workers.

## Figures and Tables

**Table 1:** Socio-economic characteristic of respondents.

Characteristic	Depression						*p*-Value
	No		Yes		Total		
	*n*	%	*n*	%	*n*	%	
Total	177	61.3	112	38.6	289	100	
Gender							
Male	22	12.6	26	23.2	48	16.7	0.02
Female	153	87.4	86	76.8	239	83.3	
Immigration status							
Local people	95	54.9	42	39.3	137	48.9	0.01
Immigrants	78	45.1	65	60.8	143	51.1	
Education							
Under high school	20	11.4	5	4.5	25	8.7	0.09
High school	101	57.7	75	67.0	176	61.3	
Above high school	54	30.9	32	28.6	86	30.0	
Marital status							
Single	7	4.0	5	4.5	12	4.2	0.83
Have spouse/partner	170	96.1	107	95.5	277	95.9	
Number of children							
0	1	0.6	11	10.3	12	4.4	<0.01
1	23	13.6	21	19.6	44	15.9	
2	112	66.3	65	60.8	177	64.1	
>2	33	19.5	10	9.4	43	15.6	
Currently living with							
Parents	83	47.4	52	46.4	135	47.04	0.87
Spouse	142	81.1	91	81.3	233	81.18	0.98
Children	108	61.7	67	59.8	175	60.98	0.75
Brothers/sisters	14	8.0	7	6.3	21	7.32	0.58
Other	1	0.74	10	7.0	11	3.96	<0.01
Annual income quintile							
1	62	36.9	34	30.9	96	34.5	0.26
2	48	28.6	42	38.2	90	32.4	
4	24	14.3	18	16.4	42	15.1	
5	34	20.2	16	14.6	50	18.0	
	Mean	SD	Mean	SD	Mean	SD	
Age	32.5	4.9	31.0	3.6	31.9	4.5	0.03
PHQ9 score	1.5	1.4	9.1	4.1	4.5	4.6	<0.01

**Table 2:** Working conditions of respondents.

Characteristic	Depression						*p*-Value
	No		Yes		Total		
	*n*	%	*n*	%	*n*	%	
Health hazards exposure at work (*n* = 278)							
Noise	122	71.8	92	84.4	214	76.7	0.02
Dust	80	47.1	60	55.1	140	50.2	0.19
High temperature	87	51.2	74	67.9	161	57.7	0.01
Toxic gas	48	28.2	57	52.3	105	37.6	<0.01
Poor lightning	11	6.5	14	12.8	25	9.0	0.07
High humidity	13	7.7	13	11.9	26	9.3	0.23
Toxic chemicals	53	31.2	47	43.1	100	35.8	0.04
Accident prone working environment	15	8.8	27	24.8	42	15.1	<0.01
	Mean	SD	Mean	SD	Mean	SD	
Years of working (*n* = 289)	9.7	3.5	10.3	4.1	9.9	3.8	0.34
Working hours per day (*n* = 275)	8.1	0.5	8.5	1.2	8.2	0.9	<0.01
Number of health hazards exposure at work (*n* = 278)	2.4	2.0	3.4	2.2	2.8	2.1	<0.01

**Table 3:** Health status and behavior of respondents.

Characteristic	Depression						*p*-Value
	No		Yes		Total		
	*n*	%	*n*	%	*n*	%	
Smoking status (*n* = 261)							
Never smoke	133	86.4	92	86.0	225	86.2	1.00
Former smokers	10	6.5	7	6.5	17	6.5	
Current smokers	11	7.1	8	7.5	19	7.3	
Hazardous drinking (*n* = 277)	19	11.2	19	17.4	38	13.7	0.14
Number of health problems (*n* = 280)							
0	39	28.5	22	15.4	61	21.8	0.05
1	36	26.3	44	30.8	80	28.6	
2	26	19.0	39	27.3	65	23.2	
>2	36	26.3	38	26.6	74	26.4	
	Mean	SD	Mean	SD	Mean	SD	
Number of health problems	1.5	1.3	2.5	1.9	1.9	1.6	<0.01

**Table 4:** Social support and community cohesion.

Characteristic	Depression						*p*-Value
	No		Yes		Total		
	*n*	%	*n*	%	*n*	%	
People in this area are willing to help each other	101	62.7	53	49.1	154	57.3	0.03
People around living place are willing to help neighbors	93	57.8	54	50.0	147	54.7	0.21
People live in harmony	97	60.3	52	48.2	149	55.4	0.05
People around can be trusted and reliable	74	46.0	43	39.8	117	43.5	0.32
People share the same value, live concept	27	16.8	15	13.9	42	15.6	0.52
This area has a tremendous amount of trash	22	13.7	25	23.2	47	17.5	0.05
This area has society’s vices	11	6.8	15	13.9	26	9.7	0.06
This area has many fights and quarrels	3	1.9	5	4.6	8	3.0	0.19
	Mean	SD	Mean	SD	Mean	SD	
Social support (score)	4.7	2.2	4.4	2.0	4.6	2.1	0.18

**Table 5:** Regression results of factors associated with depression among industrial workers.

Characteristics	PHQ9 Score		Depression	
	Coef.	95% CI	OR	95% CI
Gender (Male-ref)				
Female	−1.93	(−4.58–0.72)	0.24 ***	(0.14–0.40)
Education (Under Highschool-ref)				
Highschool	2.05 *	(−0.19–4.29)	2.51	(0.49–12.85)
Above high school	1.82	(−0.56–4.19)	2.52 ***	(1.72–3.69)
Marital status (Single-ref)				
Living with spouse/partner	−0.39	(−3.44–2.65)	1.34	(0.13–13.71)
Number of children	−1.10 **	(−1.94–−0.26)	0.47 ***	(0.27–0.82)
Local people (Yes vs No)	−0.33	(−1.49–0.82)	0.63 ***	(0.45–0.87)
Currently living with				
Parents (Yes vs No)	−0.75	(−2.00–0.51)	0.82	(0.42–1.63)
Spouse (Yes vs No)	0.41	(−1.49–2.31)	1.25	(0.47–3.33)
Children (Yes vs No)	−0.13	(−1.43–1.17)	0.93	(0.63–1.37)
Age	−0.16 **	(−0.31–−0.01)	0.92 *	(0.85–1.00)
Years of working	0.17 *	(−0.01–0.34)	1.13 ***	(1.06–1.21)
Working hours per day	0.70 **	(0.07–1.33)	1.36	(0.75–2.49)
Smoking status (Never smoke - ref)				
Former smokers	−2.02 *	(−4.43–0.39)	0.64	(0.28–1.44)
Current smokers	−2.50 *	(-5.38–0.38)	0.45 ***	(0.35–0.57)
Hazardous drinking (No-ref)				
Yes	0.48	(−2.27–3.24)	0.50 **	(0.26–0.95)
Social support (score)	−0.27 **	(−0.54–0.00)	0.92	(0.83–1.02)
Number of health hazards exposure at work	0.05	(−0.37–0.46)	1.00	(0.97–1.04)
Number of health problems	0.53 ***	(0.15–0.92)	1.37 ***	(1.24–1.52)

*** *p* < 0.01, ** *p* < 0.05, * *p* < 0.1.
